# Water-Soluble Metalated Covalent Organic Nanobelts with Improved Bioavailability for Protein Transportation

**DOI:** 10.1038/s41598-018-23744-1

**Published:** 2018-04-03

**Authors:** Weifu Kong, Jiaxun Wan, Supawadee Namuangruk, Jia Guo, Changchun Wang

**Affiliations:** 1 State Key Laboratory of Molecular Engineering of Polymers, Department of Macromolecular Science, and Laboratory of Advanced Materials, Fudan University, Shanghai, 200433 P.R. China; 2 National Nanotechnology Center (NANOTEC), National Science and Technology Development Agency, Pathumthani, 12120 Thailand

## Abstract

An available pathway to prepare the ionized covalent organic nanosheets (iCONs) has been proposed by a metal-assisted aqueous-phase exfoliation route from covalent organic frameworks. The soluble and belt-shaped iCONs could immobilize a large quantity of proteins (2.73 mg/mg, BSA/iCONs) and hence serve as transporters to enhance the protein uptake by cancer cells. Meanwhile, their energy-dependent endocytosis pathway via clathrin-coated pits has been proved as well.

## Introduction

Recent decades have witnessed enormous progress on covalent organic frameworks (COFs), which owe many of fantastic properties to their crystalline, porous and layered structures with intrinsic two-dimensional atomic frameworks^1^. Minimization of bulky COFs into the nanometer scale hold great promises for medical and diagnostic applications. Several groups have demonstrated the potent applicability of COFs in drug loading and sustained release *in vitro*^2–5^. Modification of exfoliated COFs with specific receptors also could implement the target delivery of loaded drugs^6^. In addition to shuttle cargoes, nanoscale COFs have been actively pursued as a brand-new platform for phototherapy^7^, enzyme immobilization^8^, and biomolecular detection^9^. However, the early reports have rarely investigated the fundamental interactions of COFs with living systems for improving the transportation ability of these materials. Meanwhile, such a COF-mediated cellular uptake mechanism and pathway remain, for a large part, unknown yet.

To access the cellular internalization, preparation of dispersible and discrete nanoscale COFs is a prerequisite for *in vitro* test. There are two main strategies reported thus so far for design of bioavailable COFs. One is the synthetic control over size and morphology of COF crystallite assembles that could have a diversity of forms such as microspheres, nanofibers, nanoplates and so on^10^. The other is the delamination of 2D COFs into single or few-layered covalent organic nanosheets (CONs) by destructing the relatively weak π-π interactions within neighboring layers. Comparably, the exfoliated ultrathin COFs are assumed to benefit cellular internalization because 2D organic layers could provide abundant surface and edge functionality as well as low-dimensional strength for ease of migration across cell membranes. Direct exfoliation of aromatic COFs using mechanical forces (e.g. grinding^11^ and ball milling^12^) in solid state or liquid-based ultra-sonication^13–15^ is the commonly used strategy, while such treated CONs are poorly dispersed in aqueous solution if without functional decoration. To render COFs hydrophilic, ionization of main backbones has been proposed, and the charged frameworks favor either the self-exfoliation^16^ or interfacial evolution^17^ into layered CONs. However, with this method there is an emerging need to pay more attention on challenges regarding synthesis of ionized subunits and COFs/CONs, which hence limits the development of COFs towards bio-application. Herein, we address a facile method to delamination and ionization of pristine keto-enamine-linked COFs and further immobilize fluorescence-labeled proteins for investigation of its cellular uptake mechanism.

## Results and Discussion

**Figure 1:**
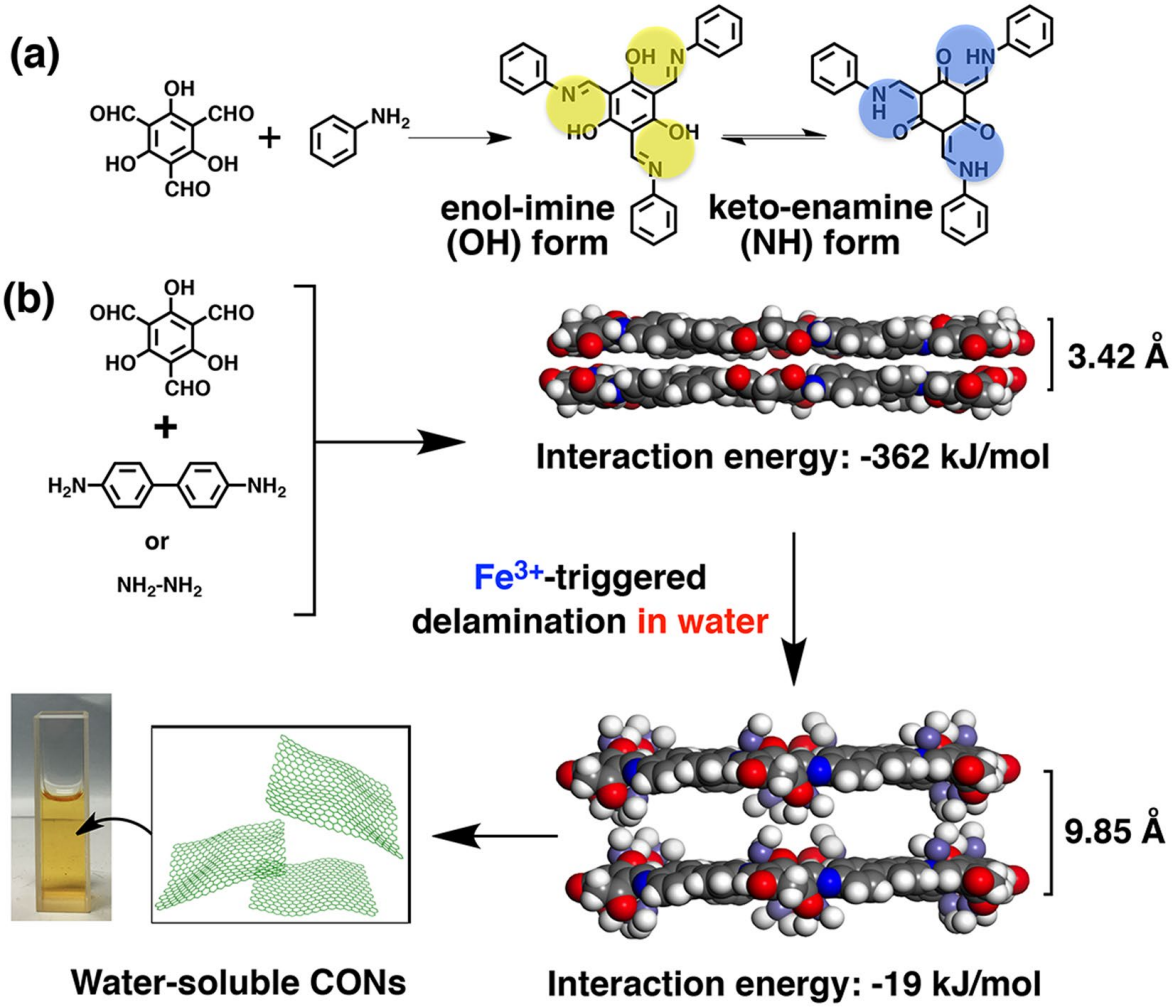
Schematic representation of metal-assisted exfoliation of keto-enamine-linked COFs in aqueous solution. (**a**) Schiff-base reaction of Tp and aniline to produce the tautomers of tris(*N*-salicylideneaniline), wherein the chelation sites are shown in circles. (**b**) Calculation of interlayer distances and interaction energies before and after the Fe(III)-assisted aqueous-phase exfoliation of COF into the corresponding soluble CON. The inset is a photograph of the dispersion of CONs in aqueous solution.

As shown in Fig. 1a, the model reaction of 1,3,5-triformylphloroglucinol (Tp) with aniline derivatives results in C_3h_ symmetric compounds that are normally considered a hybrid of keto-enamine form and enol-imine form^18^. The former tautomer has a main contribution to conjugate with metals^19^. Therefore, without incorporation of ligand subunits (e.g. phthalocyanine, porphyrin, dehydrobenzoannulene, and salen)^20–24^, a purposeful design of the COF linkers could host metals on the edges of organic networks. Then we commenced on the exfoliation of keto-enamine-linked COFs containing Fe(III)-coordinated tris(*N*-salicylideneaniline) complexes. Prior to the experimental trials, theoretical calculations have been performed by the force field method to investigate the interaction of adjacent layers upon formation of Schiff-base complexes on COF backbones. One can see that the Fe atoms with their counterpart ions reside out of plane to make the single atomic layer thicker and increase the interlayer distance (Fig. 1b). In comparison of the pristine COFs, the interaction energy between layers is largely reduced to -19 kJ/mol along with increase of the interlayer distance of up to 9.85 Å. The weak electrostatic force is assumed to maintain the superimposition of layers on each other, while such the metalated COFs should be easily intercalated by solvents to result in the independent CONs. The emerging Schiff-base complexes on backbones would largely improve hydrophilicity and hence bioavailability of such materials, which thus are accessible to functional proteins or enzymes transported into cytoplasm by using the soluble CONs as vehicles. Compared with the other known routes, metal chelation of keto-enamine-linked COFs no longer requires either the purposeful design of ionized starting materials or chemically challenging synthesis of structurally complicated ionic COFs.

Polymerization of Tp with benzidine (BD) was conducted in a mixture of *o*-dichlorobenzene and *n*-butyl alcohol (v/v, 9/1) at 120 °C for 72 h under the typical solvothermal conditions. Analogue to the early reported, the obtained products held the hexagonal lattices, high surface areas, and typical microporosity as well as insolubility. Then the COF(TpBD) powders were dispersed in water with ultrasonic assistance, while the suspension was composed of large grains even with prolonged treatment. On dissolution of FeCl_3_ salts, the suspended grains were invisible after 1-h ultra-sonication (inset in Fig. 1b), and the Tyndall effect was observed in the resulting aqueous solution, indicating that the COF aggregates were striped into discrete and dispersible nanomaterials with sufficiently small sizes out of the visible range. With removal of the free Fe(III) ions by dialysis, the homogeneity of the aqueous solution was confirmed by dynamic light scattering measurement, giving a predominated diameter of roughly 50 nm as well as narrow size distribution of PDI < 0.3 (Figure S1, Supplementary Information). By following the same treatment pathway, COF(TpHA) synthesized by Tp with hydrazine (HA), could dissolve in water as well (Figure S2, Supplementary Information). We reason that the complexation of Fe(III) ions with tri(*N*-salicylideneaniline) units of COFs occur in the 1D pore channels, and in turn, the metalated COF skeletons are ionized and hydrated leading to the disassembly of aggregates and subsequent delamination of layered structures in water. Even without mechanical forces, the electrostatic repulsion also could offset the π-π interaction of interlayers, forming dispersible nanomaterials just by letting it stand overnight. In contrast, if the imine-linked COFs evolved from the polymerization of benzene-1,3,5-tricarbaldehyde and BD, the weak coordination of -C=N- with Fe(III) ions was insufficient to allow for the metal insertion and ionization of COF skeletons for aqueous-phase exfoliation. FT IR measurement confirmed that the characteristic bands of COF(TpBD)s were maintained in the structure of exfoliated products, except of the hydrated groups around 3400 cm^−1^ (Figure S3, Supplementary Information). Also, it was found that the yield of CONs was correlated with the added amount of Fe(III) ions, and could reach >90% yields after the two runs of exfoliation procedure (Table S1, Supplementary Information).

**Figure 2:**
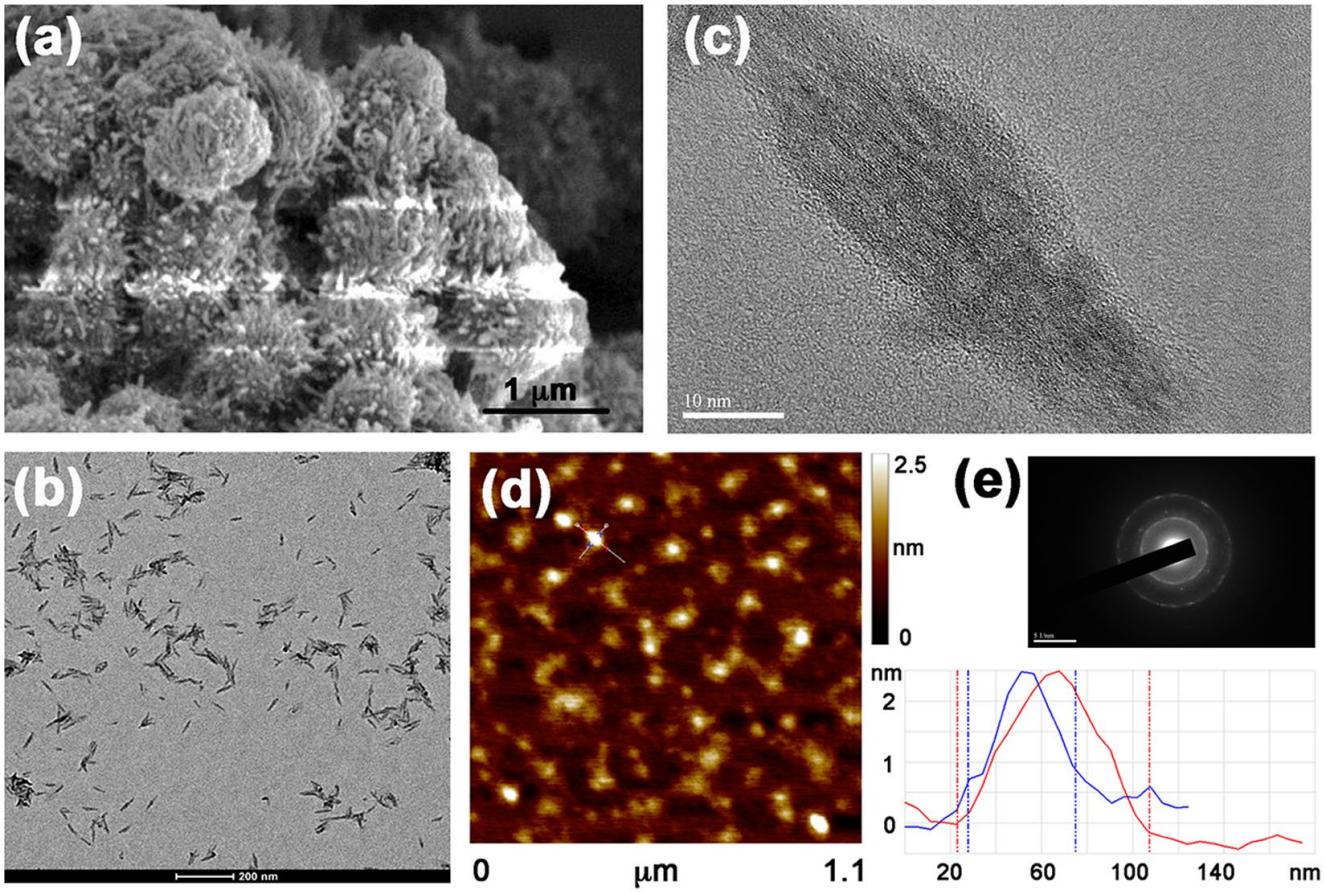
Morphology of belt-shaped CONs. (**a**–**c**) TEM image of bulk COF(TpBD) (**a**) and exfoliated CON(TpBD) (**b**) with its magnified view (**c**). (**d**) AFM image and the height profiles of exfoliated CON(TpBD). (**e**) SAED pattern of exfoliated CON(TpBD).

SEM image of  the as-synthesized COF(TpBD) in Fig. 2a reveals its clustering morphology formed with numerous nanofibers. After metal-assisted aqueous-phase exfoliation, discrete and uniform rod-like morphology is shown in TEM image, having an average length of around micron-meters (Fig. 2b). The magnification of the view on a single nanorod evidences the long-distance alignment at the molecular level (Fig. 2c and Figure S4, Supplementary Information), which is verified again by the polycrystalline diffraction rings displayed in the SAED pattern (Fig. 2e). However, the aqueous-phase XRD pattern of CON(TpBD) discloses its amorphous nature, as the main peak at 3.8° corresponding to the (100) lattice plane disappears (Figure S5, Supplementary Information). The same observation has been reported as the hydrazone-linked COFs were exfoliated^25^, implying that the delaminated CON(TpBD) retained partially ordering. To characterize the sizes of 2D geometry, AFM was performed to identify a belt-like morphology that is of a lateral length of *ca*. 80 nm and a thickness of *ca*. 2.5 nm (Fig. 2d), indicative of less than 10-layer sheets exfoliated out of the bulky COF grains. The method used here is appropriate for not only COF(TpBD), but also for the other COFs containing tri(*N*-salicylideneaniline) tautomers in the nodes of networks (Figure S6, Supplementary Information). The metal distribution was measured by the atomic mapping on the representative sample, wherein the abundant Fe atoms reside throughout the nanobelt and are overlapped with the other regions from N and O atoms (Figure S7, Supplementary Information).

**Figure 3:**
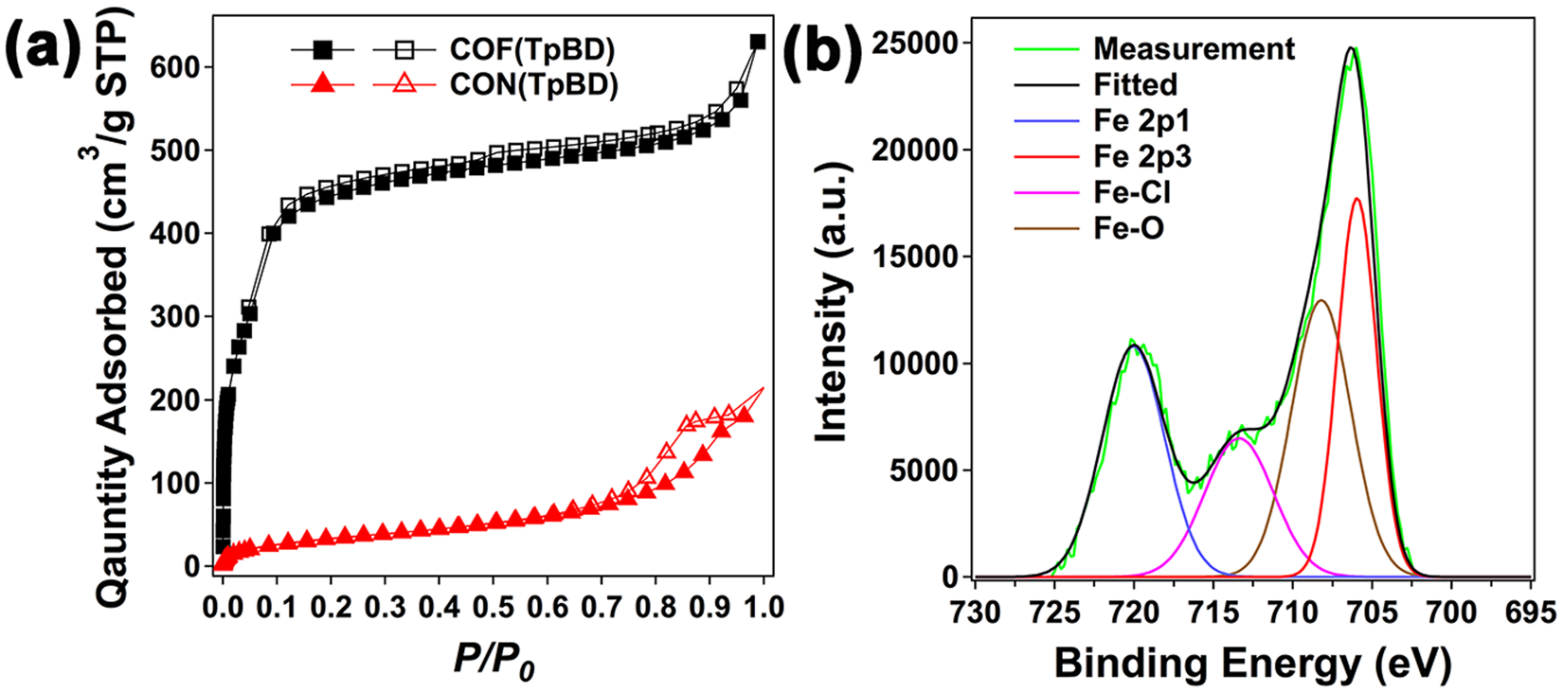
Measurements of porosity and atomic binding energy. (**a**) N_2_ sorption isotherms of COF(TpBD) and CON(TpBD). (**b**) XPS spectrum of Fe2p signal from CON(TpBD).

In addition, the nitrogen sorption isotherms (Fig. 3a) were examined to estimate the surface area by using the Brunauer–Emmett–Teller (BET) model. Compared with the pristine COF(TpBD) (1850 m^2^/g), the BET surface area of CON(TpBD) is largely reduced to be 117 m^2^/g (the detailed BET and Rouquerol plots are shown in Figures S8 and S9 in the Supplementary Information). The opening pores instead remain on the CON(TpBD), giving a broad pore-size distribution in the range of 2 to 10 nm (Figure S10, Supplementary Information).

To clarify the metalation mechanism, X-ray photoelectron spectroscopy provided insight into the binding energies of different atoms. As shown in Fig. 3b, a double broad peak from the Fe2p signal is deconvoluted into four individual peaks at 720 eV, 706 eV, 713 eV, and 708 eV, which are assignable to Fe2p1, Fe2p3, Fe-Cl, and Fe-O units, respectively. This reveals that the dominant keto-enamine structures^26^ could conjugate with Fe(III) ions in HN···O pockets. On the other hand, it was detected that 26 wt.% of Fe atoms is immobilized into the CONs. The calculated molar ratio of Fe and tri(N-salicylideneaniline) units is close to 3:1, indicating the formation of trinuclear Fe(III) complexes. For extension of the available metal species, Fe(II), Zn(II) and Cu(II) ions were testified to exfoliate the keto-enamine-linked COFs, whereas they didn’t work with the identical operation. We reason that some kinds of the metal complexes formed on COF skeletons cannot attenuate the π-π interaction between the neighboring layers.

**Figure 4:**
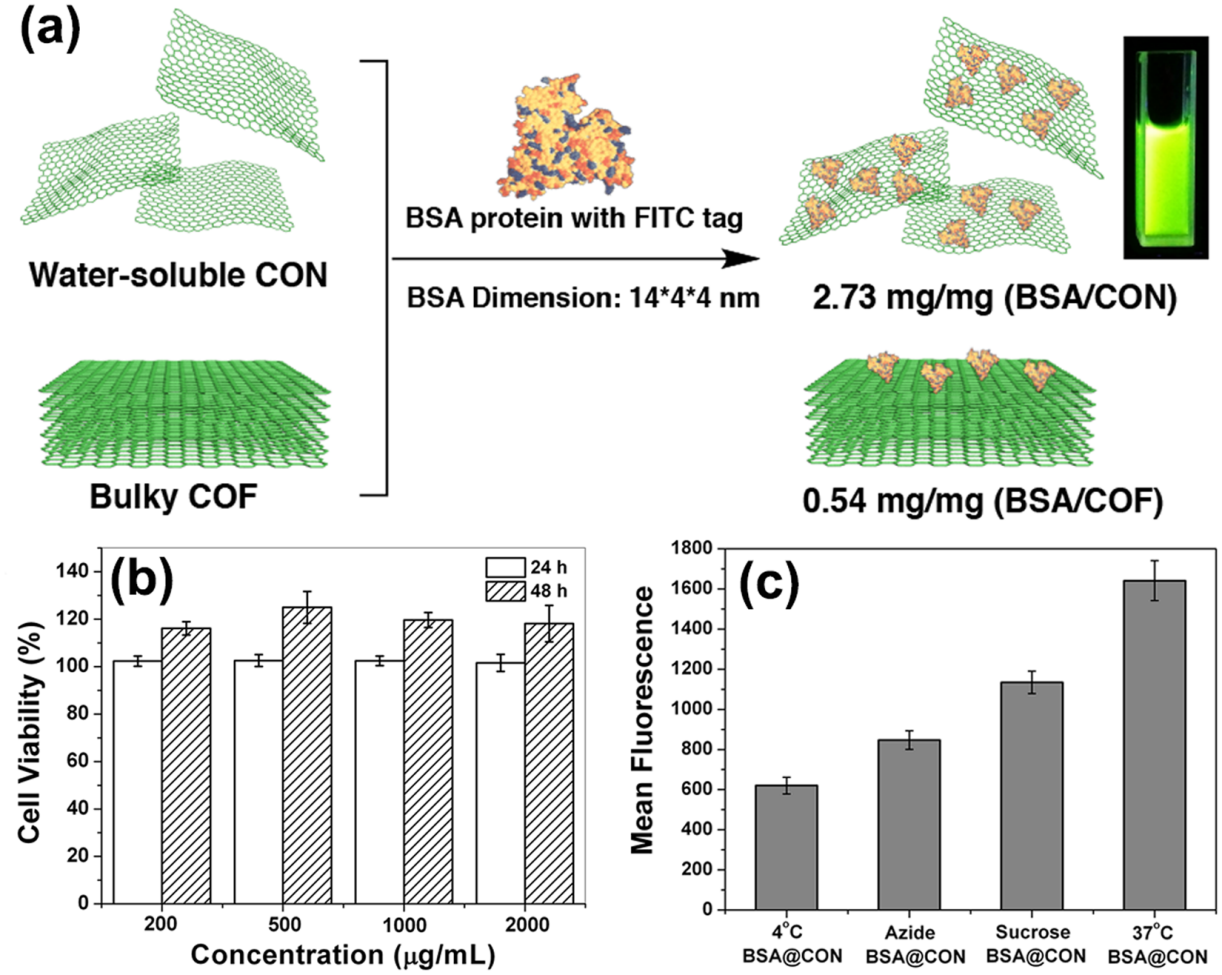
Loading and transportation of FITC-labeled BSA proteins with non-cytotoxic CONs. (**a**) Schematic representation of loading BSA-FITC proteins on CONs and COFs, and the fluorescent image shows the dispersion of FITC-BSA@CON in PBS solution under UV lamp. (**b**) Cytotoxicity test of CON(TpBD) incubated with Hep G2 cells for 24 h and 48 h, respectively. (**c**) Flow cell cytometry data of Hep G2 cells after incubation in FITC-BSA@CON conjugates with pretreatment at 4 °C or with NaN_3_ or sucrose. The regular group was incubated at 37 °C without any pretreatment.

Prior to the study of intracellular uptake pathway for the protein-loaded CONs, we estimated the cytotoxicity and intracellular distribution of CON(TpBD) in Hep G2 cancer cells. As observed in Fig. 4b, nearly no Hep G2 cells are dead at the maximum concentration of 2000 μg/mL, and over 100% viability could be maintained after 48 h. To access the intracellular distribution of transporters, the residual amino groups on the periphery of CONs were employed to react with fluorescein isothiocyanate (FITC) to label the CON materials. The FITC-modified CON shows a strong fluorescence emission around 513 nm with an excitation wavelength of 490 nm (Figure S11, Supplementary Information). After a 2-h incubation with Hep G2 cells, the CON probes accumulate within cells, and the produced green fluorescence is mainly distributed around the cell nucleus (Figure S12, Supplementary Information).

In view of metalated and porous interfaces of the soluble CONs, FITC-labeled BSA proteins could be readily adsorbed on it by the electrostatic interaction. The aqueous dispersion of FITC-BSA@CONs displays brilliant green fluorescence with UV lamp irradiation (Fig. 4a). The loaded BSA protein reaches as high as 2.73 mg per milligram of CONs in PBS solution evaluated by the standard curve of FITC-BSA (Figure S13, Supplementary Information). To our knowledge, the protein-loading efficiency of CONs is much higher than those of the analogous 2D or porous materials such as oxidized graphene (1.5–1.8 mg/mg)^27–29^, and MOFs (0.18 mg/mg)^30^. In sharp contrast, the bulky COF powders have the relatively low loading capacity (0.54 mg/mg, BSA/COF), mainly because the BSA protein has so large dimension that nanopores of COFs (1~3 nm) are incapable of immobilizing them.

Next, we underwent a rigorous investigation of the cellular internalization mechanism and pathway for CON-based conjugates. In general, low-molecular-weight solutes transport through the plasma membrane via the energy-independent pathway. Relatively large particles are internalized by the energy-dependent pathway, also known as endocytosis that could be hindered when incubation are performed at 4 °C or in adenosine triphosphate (ATP)-free medium^31^. ATP production in cells can be disturbed with NaN_3_, thus blocking the endocytotic pathway. Along this line, Hep G2 cellular incubations with FITC-BSA@CON conjugates were carried out at 4 °C and with the cells pretreated with NaN_3_ for 1.5 h, respectively, and the cells incubated at 37 °C without any pre-treatment were used as a control group. Indeed, a low fluorescence level detected by flow cytometry measurement was obtained for the cells incubated at 4 °C and in the presence of NaN_3_, respectively. This indicates endocytosis as the internalization mechanism for the uptake of BSA-CON at 37 °C. Then we studied the subcategories of the endocytosis pathway, which usually refer to phagocytosis, pinocytosis, and clathrin-dependent receptor-mediated and clathrin-independent endocytosis^32^. Of varieties, the most common pathway is receptor-mediated endocytosis, which occurs when the clathrin coat on the plasma membrane forms invaginations in the membrane to trap the extracellular species^33^. To access the role of clathrin in the uptake of CON conjugates, hypertonic treatment with sucrose was carried out to disrupt the formation of clathrin-coated vesicles on the cell membrane. It was observed that the fluorescence reduced when the cells were pretreated with sucrose, suggesting that the entry of BSA@CON conjugates into Hep G2 cells is mainly through a clathrin-dependent endocytosis pathway. However, the protein-CON conjugates was observed inside the cells even at 4 °C or in the presence of NaN_3_, which indicates that CON can partly enter into cells directly through an energy-independent pathway involving insertion and diffusion across the cell membrane. The enhanced internalization ability implies the superiority of low-dimensional CON as protein transporter.

## Conclusion

In summary, metalated covalent organic nanosheets with few-layer molecular thickness is prepared by an aqueous-phase exfoliation method, which has been proved facile, efficient, and eco-friendly for disassembly of the pristine keto-enamine-linked COFs. The obtained CONs display uniform belt shape, water solubility and metal coordination as well as partial periodicity and porosity in structure. The bioavailability of CONs has been substantially proved by its non-cytotoxicity and enhanced loading capacity of proteins. We further have demonstrated that these ionized CONs are capable of the transportation of proteins into living cells and that the cellular-uptake mechanism is energy-dependent endocytosis, which occurs mainly through clathrin-coated pits. Establishment of the entry mechanism is of fundamental importance and will facilitate future developments of COF-related transporters for biological delivery applications.

## Materials and Methods

### Materials

1,3,5-Trihydroxybenzene (THB) was purchased from Adamas Reagent Company. Trifluoroacetic acid (TFA), benzidine (BD), pyrrolidine, hydrazine hydrate, *o*-dichlorobenzene (DCB), fluorescein isothiocyanat (FITC) and *n*-butanol were purchased from Aladdin Industrial Corporation. Hexamethylenetetramine (HMT), acetic acid (HOAc), iron(III) chloride hexahydrate (FeCl_3_•6H_2_O), and acetone were purchased from Shanghai Chemical Reagents Company. All reagents were purchased and used as received. Deionized water was used in all experiments.

### Synthesis of 1,3,5-triformylphloroglucinol

According to a modified method presented by Foster *et al*.^34^, hexa-methylenetetramine (15.10 g, 108 mmol) and phloroglucinol were added to 90 mL of trifluoroacetic acid under N_2_ atmosphere, and the solution was heated to 100 °C and maintained for 2.5 h. Then 150 mL of 3 M HCl aqueous solution was mixed to allow for another 1-h reaction at 100 °C. After being cooled to room temperature, the mixture was filtered through Celite, extracted with ca. 350 mL dichloromethane, and dried over MgSO_4_. After rotary evaporation, 4.19 g off-white powder was obtained in 19% yield. ^1^H NMR (400 MHz, CDCl_3_): δ (ppm) 14.12 (−CHO, s, 1 H), 10.18 (−OH, s).

### Synthesis of keto-enamine-linked COFs

According to the typical solvothermal protocol^26,35^, the keto-enamine-linked COFs were synthesized in a sealed vial, wherein 1,3,5-triformylphloroglucinol (10.5 mg, 0.05 mmol) and benzidine (13.8 mg, 0.075 mmol) were partially dissolved in a mixture of o-dichlorobenzene and n-butyl alcohol (v/v, 9:1, 1 mL) with addition of the aqueous solution of acetic acid (6 M, 0.1 mL). The reaction mixture was subjected to a freeze-pump-thaw process for 3 cycles, and the sealed vial was kept in an oven at 120 °C for 72 h. After reaction, the solids were washed by acetone for three times in Buchner funnel. The target COF(TpBD) powders were obtained in 75% yield. Following the same steps, the COF(TpHA) solids were also obtained in 70% yield, using 1,3,5-triformylphloroglucinol (10.5 mg, 0.05 mmol) and hydrazine hydrate (3.25 μL, 0.075 mmol).

### Preparation and modification of CONs

The solid COF(TpBD) (2 mg) was added in the aqueous solution of FeCl_3_ salt (3.8 mg, 4 mL). The COF grains were initially suspended in solution. After sonication for 1 h, they could be gradually dissolved, and the solution turned transparent and yellow colour. The residual solids were then collected by centrifugation and subjected to the same treatment in a fresh FeCl_3_ aqueous solution (4 mL). The obtained CON solutions were combined to eliminate the free iron salts by dialysis, and freeze-dried to obtain the yellowish powder with >90% yield. To modify the obtained CON(TpBD)s with fluorescent dyes, the isothiocyanate group of FITC molecules were reacted with the residual amino groups located on the periphery of nanosheets in ethanol at 70 °C overnight. After reaction, the free FITC was removed by dialysis in ethanol, and the aqueous dispersion of FITC-modified CON(TpBD) was freeze-dried for further use.

### Theoretical calculation

Compass (condensed-phase optimized molecular potentials for atomistic simulation studies)^36^ force-field included in the Forcite module of the Material Studio package was used to calculate the interlayer distances and interaction energies (${E}_{{int}}$) between the two adjacent layers of COF before and after insertion of Fe(III) ions and complexation. The systems were optimized in gas phase without any constraint. Convergence criteria of all the calculations were determined according to the ultrafine settings of the program. Partial charges on the atoms were conducted by the force field assigned charging methods that was included in the Compass force fields. The interaction energy of the two neighboring layers is expressed by

1 $${E}_{int}={E}_{2COF}-2{E}_{COF}$$

where ${E}_{2{COF}}$ and ${E}_{{COF}}$ are both the total energies of optimized interlayers and single layer of COF, respectively. The large negative value means the strong interaction between two layers. The interlayer distance of COF was measured from the distance between the centroid of each layer.

### Cell culture

Hep G2 cells were cultured in DMEM (high glucose) medium supplemented with 10% fetal bovine serum (FBS) with 100 U/mL penicillin and 0.1 mg/mL streptomycin at 37 °C in a humidified 5% CO_2_ incubator (HERAcell 150i). Subculture was performed every two days; for general cell culture, 25 cm^2^ of cell culture flask was used. Briefly, cells were digested using 0.25% trypsin (1 mL) containing 0.02% EDTA solution at 37 °C for 3 min, then complete cell culture medium (2 mL) was added and cells were gently piped off at bottom of culture flask. Cell suspensions were centrifuged at 100 g for 3 min, the supernatant was discarded, and the complete culture medium (3 mL) was added and piped to obtain a single cell suspension. Cells suspension (1 mL) was transferred to a new 25-cm^2^ cell culture flask, and the complete culture medium (4 mL) was added to re-suspend cells. After that, it was placed in CO_2_ cell incubator for further culture.

### Cell viability test

Hep G2 cells were seeded in 96-well plates at the density of 5000 cells per well, wherein volumes of culture medium were all kept at 100 μL. After being incubated for 24 h, culture medium was replaced with those medium containing the dispersion of CON(TpBD). The cell viability at 24 h and 48 h were determined by CCK8 assay, respectively.

### Cellular uptake of CON-based samples

Hep G2 cells were seeded in 6-well culture dish at 2 × 10^5^ cells per well for 24-h incubation at 37 °C. Then BSA-FITC@CON was dispersed in PBS buffer to keep the concentration at 20 μg/mL. Prior to the cultivation of samples with cells, pretreatment of the cells was carried out as follows: (1) the culture medium kept at 4 °C, instead of the regular 37 °C condition; (2) 10 mM NaN_3_ in PBS buffer was added to treat the cells for 30 min at 37 °C, in order to reduce the intracellular ATP level; (3) 0.45 M sucrose in PBS buffer was added to treat the cells for 30 min at 37 °C, in order to disrupt the formation of clathrin-coated vesicles. After that, the mixture in the presence of BSA-FITC@CON was incubated for 1.5 h. The cell culture medium was then discarded, and the residues were washed with PBS for three times to remove the remaining BSA-FITC@CON. Cells were digested and centrifuged, and then analyzed by the flow cytometry measurement data.

### Characterization

Detailed morphological and structural characterizations were carried out by using high-resolution transmission electron microscope (HR TEM) (JEOL 2100 F, Japan), operated at 200 kV accelerating voltage at room temperature. The elemental mappings of Fe, N, and O atoms were collected using the same transmission electron microscope (operating at 200 kV) under the ADF STEM mode. FT IR spectra were recorded on Nicolet 6700 (Thermofisher, USA) Fourier transformation infrared spectrometer. Samples were dried and mixed with KBr to be compressed to a plate for measurement. ^1^H NMR spectra were recorded on Varian Mercury plus 400 MHz spectrophotometer at 298 K. Solution-phase X-ray diffraction (PXRD) patterns were collected on an X-ray diffraction spectrometer (Bruker D8 Advance, Germany) with Cu Kα radiation at λ = 0.154 nm operating at 40 kV and 40 mA. Hydrodynamic diameters were conducted with a Nano ZS Zetasizer (model ZEN3600, Malvern Instruments) using a He–Ne laser at a wavelength of 632.8 nm. Fluorescence spectra were obtained on a FLS920 spectrometer (Edinburgh Instruments). The morphology and height image of samples were obtained by scanning probe microscope (Bruker, Multimode 8). Flow cytometry measurement was carried out by flow cytometer (Beckman Coulter, A41). N_2_ adsorption-desorption isotherms were collected by a TriStar II 3020 volumetric adsorption analyzer (Micromeritics, USA) at 77 K. The samples were degassed at 200 °C for 24 h before measurement. Pore-size distribution was analyzed by non-local density functional theory (NLDFT) with N_2_-Carbon kernel based on a slit-pore model.

## Electronic supplementary material

Supplementary Information
