# Supplementary material for: Water-Soluble Metalated Covalent Organic Nanobelts with Improved Bioavailability for Protein Transportation
Source: Sci Rep. 2018 Apr 3;8:5529. doi: 10.1038/s41598-018-23744-1 (PMC5883060; doi:10.1038/s41598-018-23744-1)
Supplement: Supplementary file 1 — Supplementary Information [file 41598_2018_23744_MOESM1_ESM.pdf]

Supplementary Information

Water-Soluble Metalated Covalent Organic Nanobelts with Improved Bioavailability for Protein Transportation

Weifu Kong,[1] Jiaxun Wan,[1] Supawadee Namuangruk,[2] Jia Guo[1,*] & Changchun Wang[1]

[1] State Key Laboratory of Molecular Engineering of Polymers, Department of Macromolecular Science, and Laboratory of Advanced Materials, Fudan University, Shanghai 200433, P. R. China

[2] National Nanotechnology Center (NANOTEC), National Science and Technology Development Agency, Pathumthani 12120, Thailand

* E-mail: guojia@fudan.edu.cn

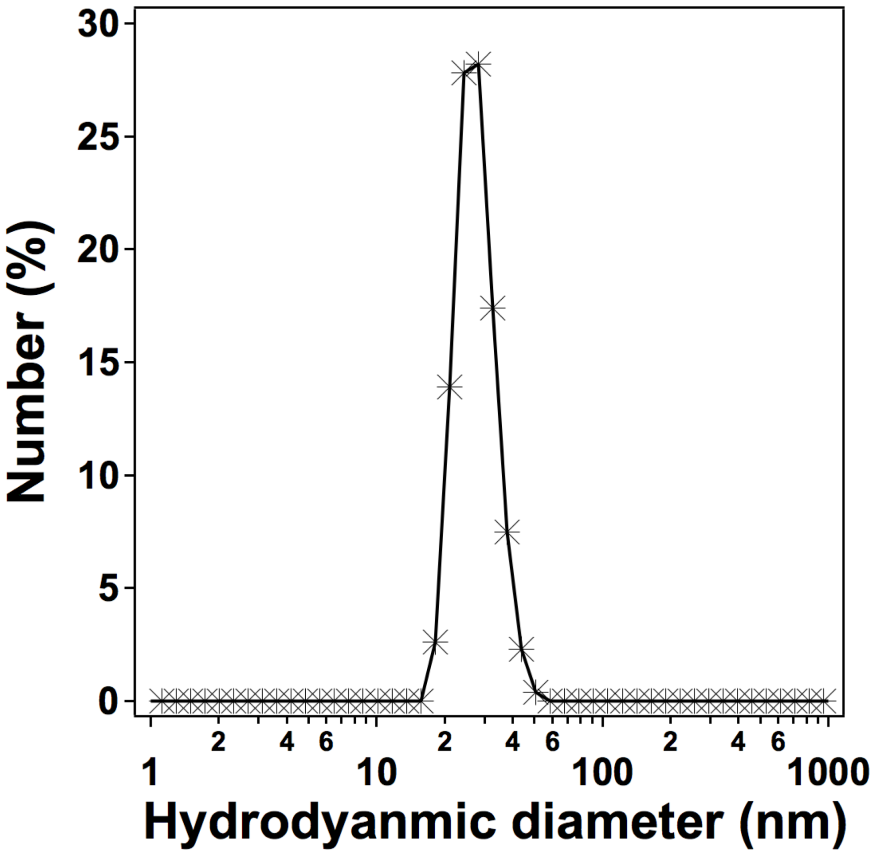

Figure S1. Dynamic light scattering of the aqueous dispersion of CON(TpBD) exfoliated from the corresponding COF in water.

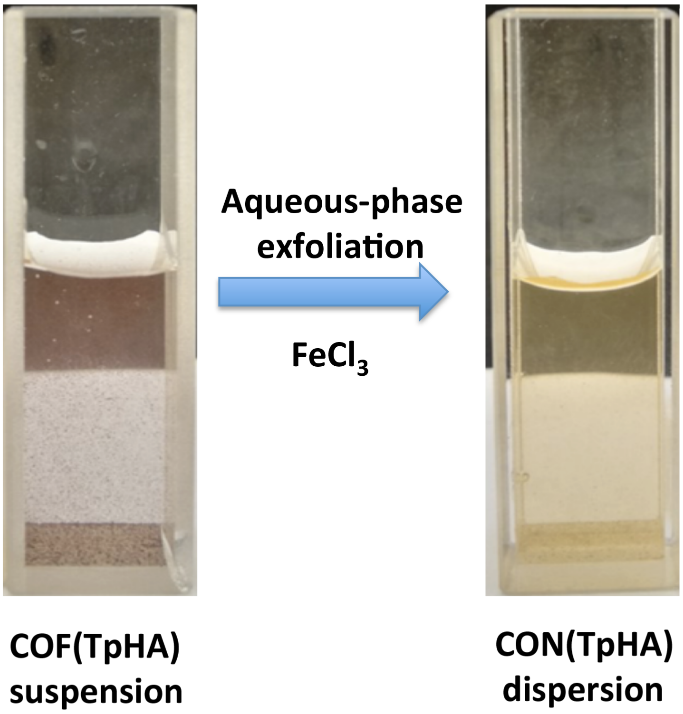

Figure S2. Metal-assisted aqueous-phase exfoliation of bulk COF(TpHA) solids with addition of $FeCl_3$ salts under sonication.

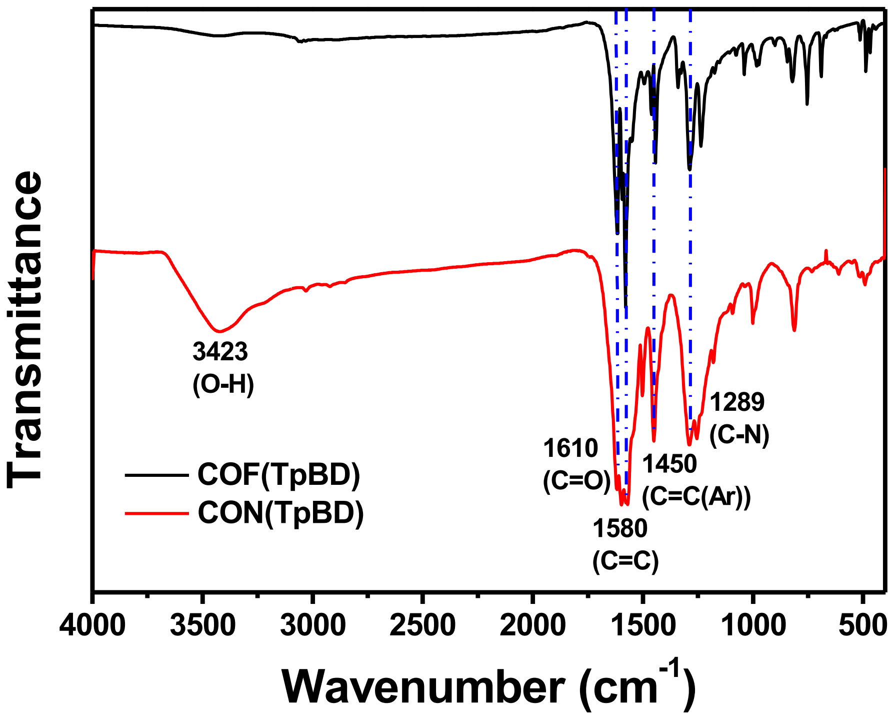

Figure S3. FT IR spectra of the bulk COF(TpBD) and exfoliated CON(TpBD), respectively.

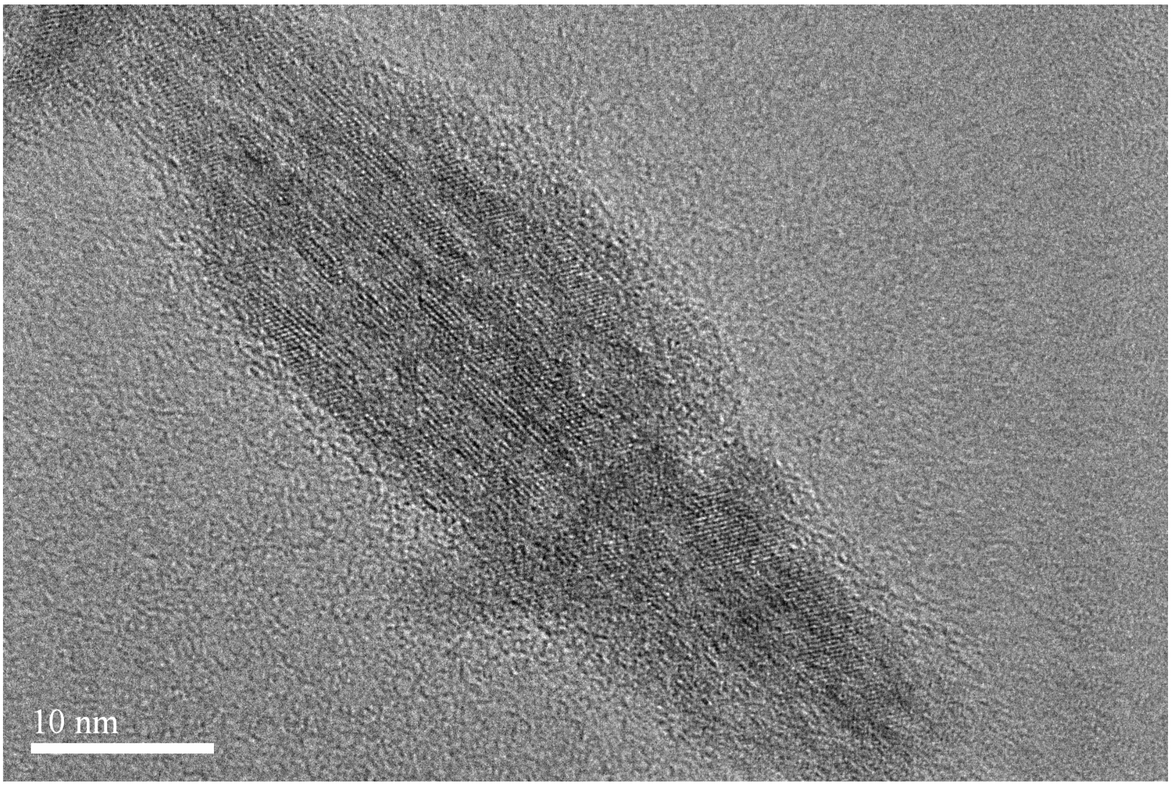

Figure S4. HR TEM image of the CON(TpBD) nanobelts displaying the long-range ordering lattice structure.

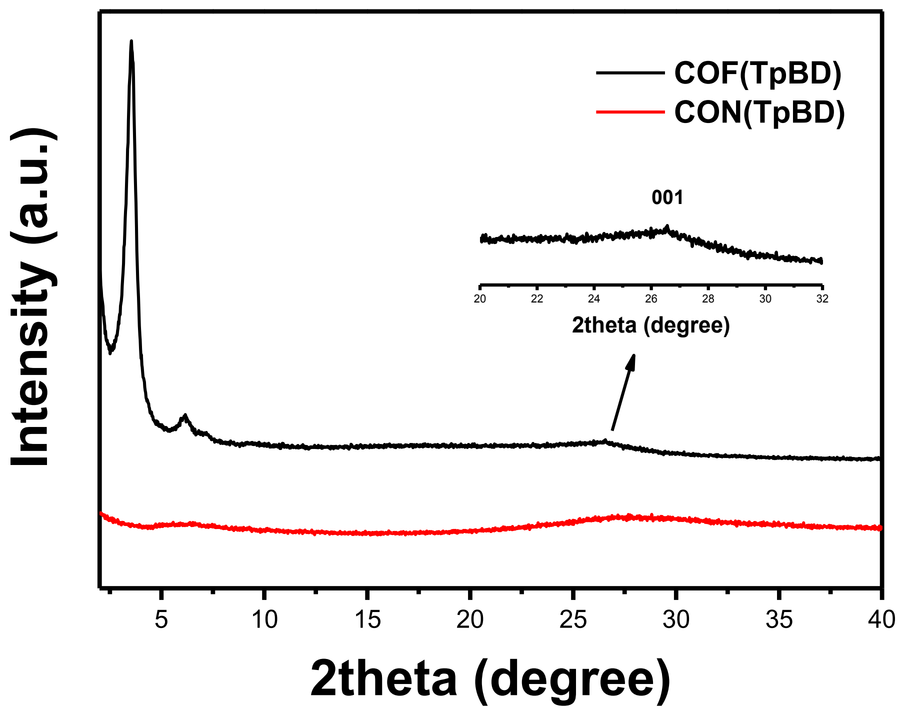

Figure S5. XRD patterns of COF(TpBD) and CON(TpBD), respectively. The inset is a magnified view of the COF(TpBD) pattern in the angle range from 20° to 32°.

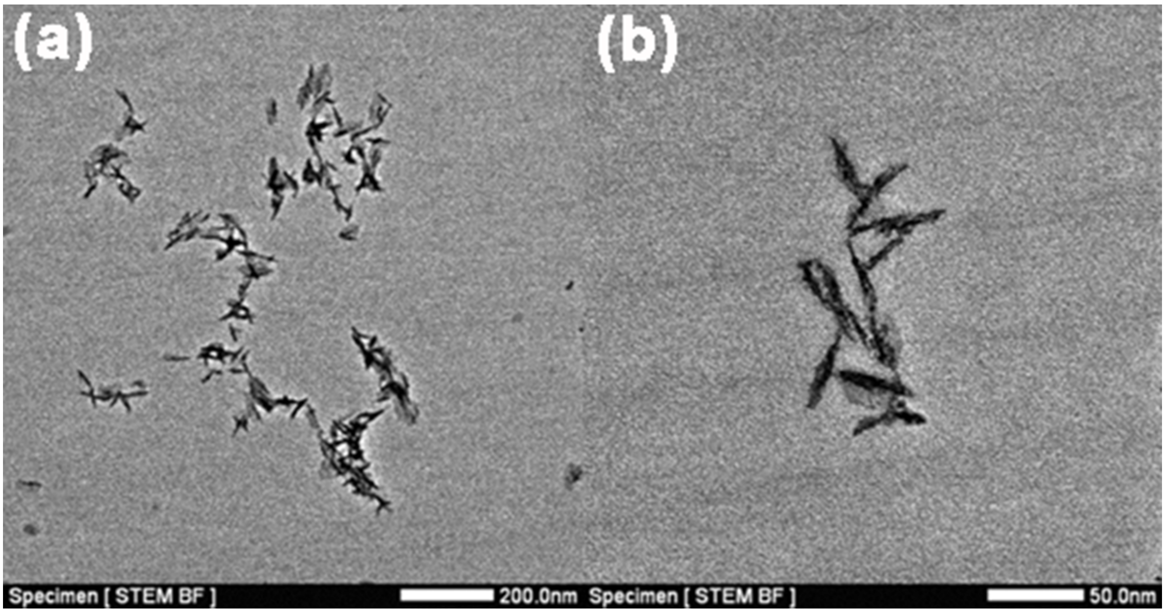

Figure S6. TEM image (a,b) of the exfoliated CON(TpHA) exhibiting the uniform rod-like morphology with an average length of 50 nm.

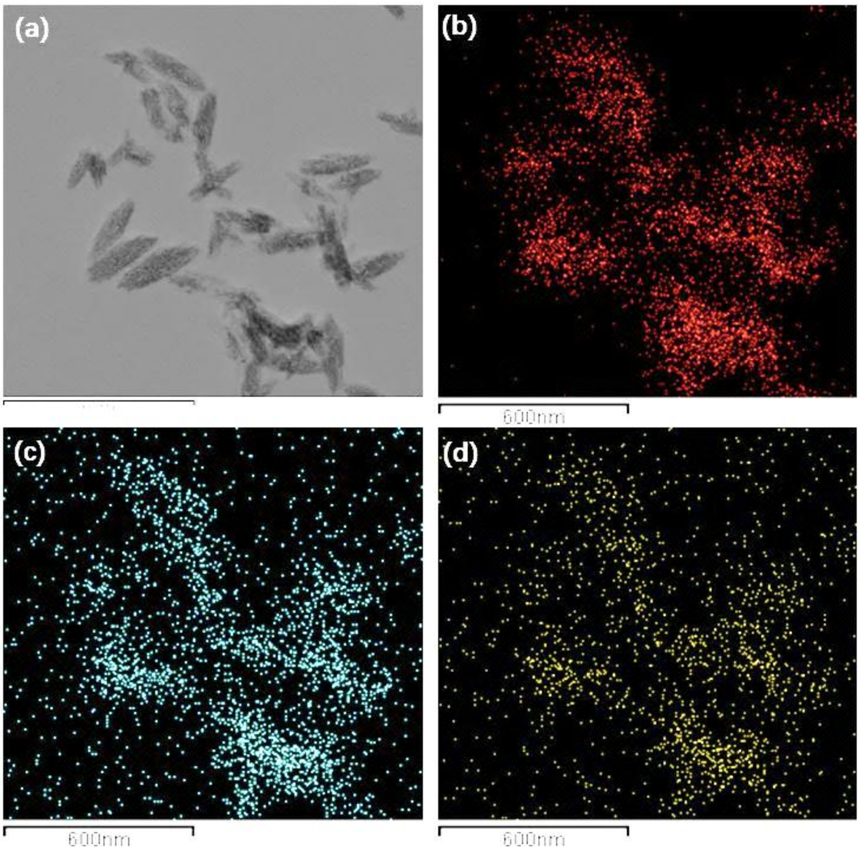

Figure S7. TEM image (a) and elemental mappings (c-d) of Fe (b), O (c), and N (d) atoms obtained on the exfoliated CON(TpBD)s with conjugation of Fe(III) ions.

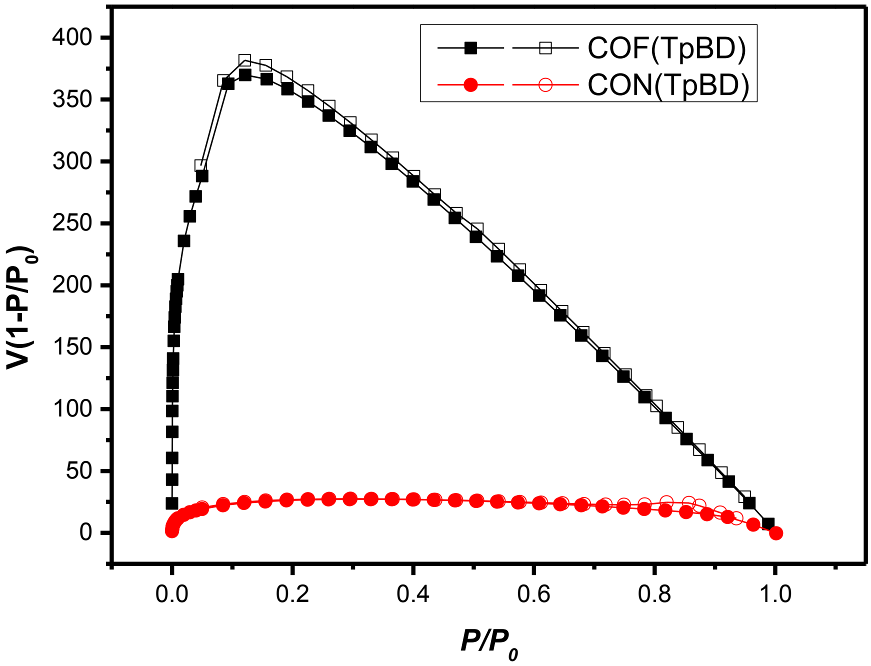

Figure S8. Calculated Rouquerol plot of COF(TpBD) and CON(TpBD), respectively.

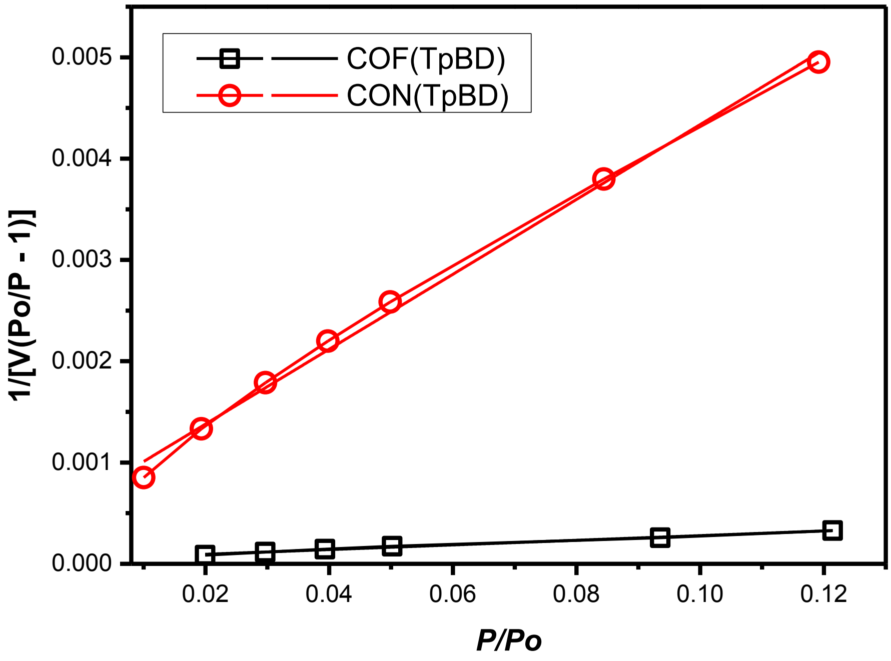

Figure S9. BET plots of COF(TpBD) and CON(TpBD), whose Constant C are calculated to be 48.7 and 58.7, respectively.

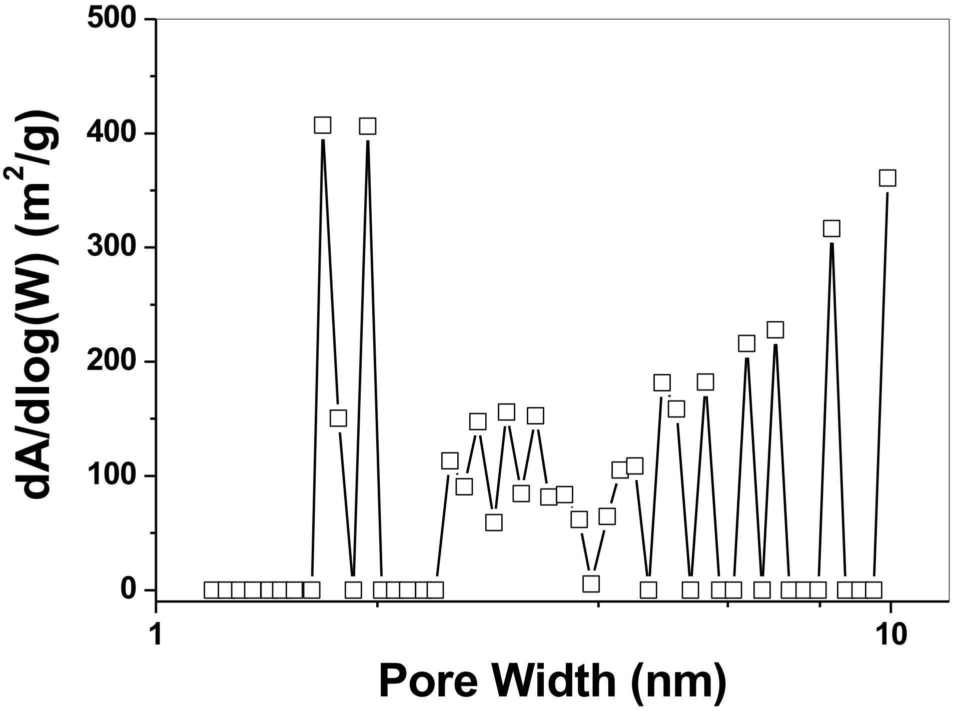

Figure S10. Pore-size distribution of CON(TpBD) calculated by NLDFT model.

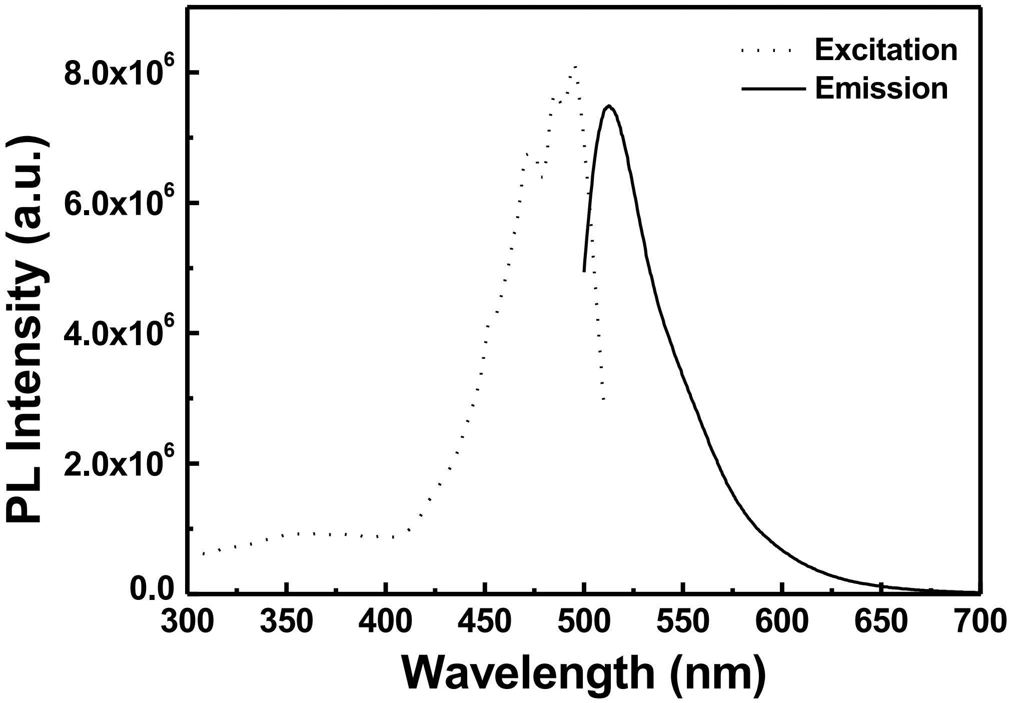

Figure S11. Fluorescence excitation (dot) and emission (line) spectra of the FITC-modified CON(TpBD).

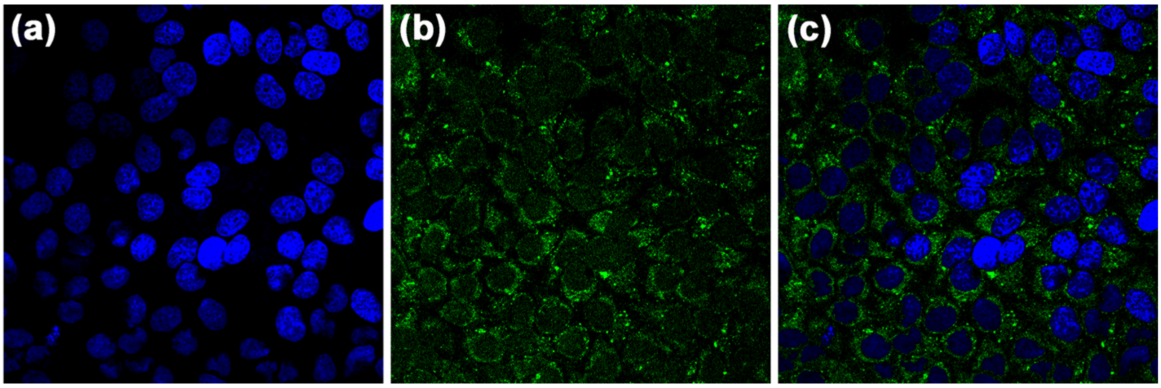

Figure S12. Fluorescence images of Hep G2 cells labelled with 4,6-diamidino-2-phenylindole (a) and FITC-modified CON(TpBD) (b), with their overlaid image (c).

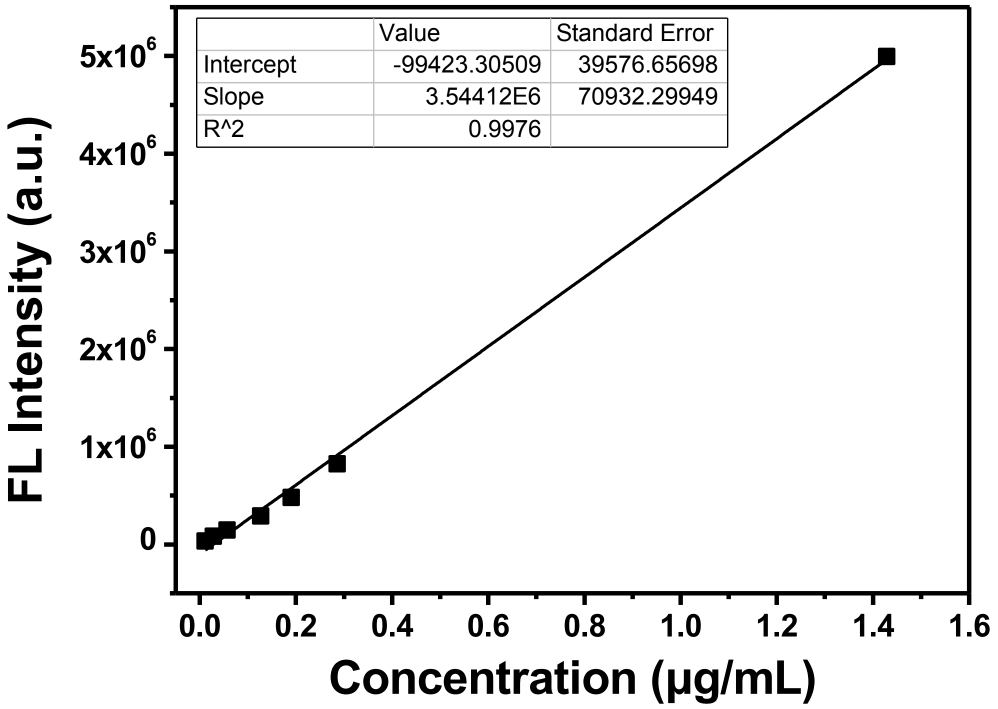

Figure S13. The standard curve of BSA-FITC concentration *vs.* fluorescence intensity in aqueous solution.

Table S1. Exfoliation yields for COF(TpBD)s using the different concentrations of $FeCl_3$ salts.

COF(TpBD) (mg)	$FeCl_3$ (mg)	H_2O (mL)	Yield (%)
2.0	0.6	4	29.4[a]
2.0	1.3	4	43.5[a]
2.0	3.8	4	64.0[a]<br>>90[b]

[a] To determine the exfoliation yield, the un-exfoliated solids collected by centrifugation were dried and weighed to calculate the yield of soluble CONs through the comparison of the feeding amount of COFs. [b] After the exfoliation of COFs, the residual solids in solution were collected by centrifugation and subjected to the same treatment again. With removal of free salts by dialysis, the CONs obtained over two runs were freeze-dried and combined to calculate the total yield.
